# Antibiotic Misuse Behaviours of Older People: Confirmation of the Factor Structure of the Antibiotic Use Questionnaire

**DOI:** 10.3390/antibiotics12040718

**Published:** 2023-04-06

**Authors:** Loni Schramm, Mitchell K. Byrne, Taylor Sweetnam

**Affiliations:** Faculty of Health, Charles Darwin University, Darwin, NT 0909, Australia

**Keywords:** antibiotic misuse, older adults, Theory of Planned Behaviour, antimicrobial resistance, antibiotic stewardship

## Abstract

Antibacterial resistance (AR) is responsible for steadily rising numbers of untreatable bacterial infections, most prevalently found in the older adult (OA) population due to age-related physical and cognitive deterioration, more frequent and long-lasting hospital visits, and reduced immunity. There are currently no established measures of antibiotic use behaviours for older adults, and theory-informed approaches to identifying the drivers of antibiotic use in older adults are lacking in the literature. The objective of this study was to identify predictors of antibiotic use and misuse in older adults using the Antibiotic Use Questionnaire (AUQ), a measure informed by the factors of the Theory of Planned Behaviour (TPB): attitudes and beliefs, social norms, perceived behavioural control, behaviour, and a covariate—knowledge. A measure of social desirability was included, and participants scoring highly were excluded to control for social desirability bias. Confirmatory Factor Analyses and regression analyses were conducted to test the hypotheses in a cross-sectional, anonymous survey. A total of 211 participants completed the survey, 47 of which were excluded due to incompletion and high social desirability scores (≥5). Results of the factor analysis confirmed that some (but not all) factors from previous research in the general population were confirmed in the OA sample. No factors were found to be significant predictors of antibiotic use behaviour. Several suggestions for the variance in results from that of the first study are suggested, including challenges with meeting requirement for statistical power. The paper concludes that further research is required to determine the validity of the AUQ in an older adult population.

## 1. Introduction

### 1.1. Driving Factors of Antibiotic Resistance

Antimicrobial resistance (AMR), and in particular, antibiotic resistance (AR), is a growing concern in the health service provision [1]. AMR is described as the gradual changing of organisms—such as bacteria, viruses, fungi, and parasites—such that they become resistant to medicines and make infections harder to treat. These resistant organisms contribute to the spread of disease and chronic illness, and increase the risk of death [2]. There are heightened concerns around AR due to the apparent overuse of antibiotics in agricultural and medical settings [3]. The indiscriminate use of antibiotics is thought to be a driver of AR as bacterium develop defenses against antibiotics, resulting in a loss of efficiency in disease treatment [4].

AR contributes to increased mortality globally and is estimated to result in approximately 1600 Australian deaths annually [5]. As antibiotics become less effective, more infections will require the use of increasingly limited medical treatments or simply be untreatable [2]. The risk of AR infections and AR-related deaths is disproportionately higher for older adults due to their increased susceptibility to age-related comorbidities, making this population a high priority when conducting research on antibiotic use behaviours [6,7].

Currently, Australia’s National Antimicrobial Resistance Strategy (NARS) has implemented multiple interventions into Australian healthcare systems in an attempt to reduce rates of unnecessary antibiotic use and increasing medical literacy amongst pharmacists and GPs [5,8]. Still, antibiotic use in Australia ranks highly amongst other wealthy countries, with prescribing rates in children approximately 30% higher than in the USA, and twice as high in adults (per capita) than Sweden [9,10]. Antibiotics are still frequently prescribed inappropriately for reasons unaligned with clinical practice guidelines. For example, 81% of Australian patients in 2017 received antibiotic prescriptions for upper respiratory tract infections, for which antibiotics are not recommended [1,11]. While prescriptions for antibiotics are decreasing annually, in 2019 over 26 million prescriptions were dispensed by GPs to at least 40.3% Australians [1]. Drivers of antibiotic misuse within the community include a lack of public health literacy and knowledge of AR/AMR, accessibility to non-prescribed antibiotics, and the level of stewardship involving healthcare professionals [4].

### 1.2. Public Health Literacy of AR and Prevalence of Antibiotic Use in Older Adults

Health literacy describes the skills and knowledge of a person regarding their health and healthcare systems [12]. It includes their ability to locate, interpret, and communicate health-related information, and use their knowledge of health services to seek appropriate care [12]. Lack of knowledge and awareness is a large contributor to the misuse of antibiotics and is predominantly determined by both education level and accessibility to public information [10]. In Machowski and Stålsby-Lundberg’s (2019) review, 57% of Europeans in the general population were unaware of antibiotic ineffectiveness against viruses, 44% were unaware of ineffectiveness against colds and influenza, and approximately 20% considered it unlikely that AR would affect them or their family. The most common misconception regarding AR among older adults was that only humans (and not bacteria) become resistant to antibiotics with prolonged use, and therefore they would not contribute to AR [10]. Overall, older adults were more likely to overestimate their AR knowledge, with the belief that having previously taken specific antibiotics for familiar symptoms meant they could take them again—with or without a prescription [13,14]. Demographic predictors for antibiotic use behaviour varied by country: for some, use was reported as 7% higher for those less educated and 13% higher for those in worse economic circumstances, while other countries showed the opposite, with higher antibiotic use in higher-income families [15]. These findings suggest that population-specific health education strategies are essential for AR-focused interventions [7].

Older adults’ health anxiety and health needs surpass those of younger people, and the incidence of GPs wrongly prescribing antibiotics is more frequent for older adults [16,17]. It is therefore particularly important to measure levels of health literacy and its influence on antibiotic use behaviours in this population [6]. Common health conditions frequently misconceived by older adults as requiring a prescription for antibiotics include upper respiratory tract infections, urinary tract infections, seeking relief from pain symptoms, and common colds and flu [18,19,20]. These misconceptions are likely driven from fear of an increased risk to health, and worsening age-related health issues [21]. Compared to younger adult age groups, clinical presentations of atypical infections, rapid disease progression, risk of inappropriate treatment, and prolonged recovery periods are more common in older adults, whose risk of exposure to AR is heightened if they live alone with limited access to health information [22,23].

### 1.3. Antibiotic Misuse and Stewardship in Older Adults

According to the World Health Organization (WHO) (2021), inappropriate use of antibiotics occurs when they are obtained or prescribed without appropriate diagnosis as treatment for symptoms not included in the health guidelines, in doses that are excessive (i.e., with treatment courses longer than the infection requires), with unnecessary repeat prescriptions, and/or when antibiotic treatment information and risks are not adequately communicated to the patient. Non-prescription antibiotic use, non-adherence to antibiotic use guidelines, and antibiotic hoarding are classified as misuse of antibiotics [24]. Despite the WHO declaring antibiotics a prescription-only medicine and limiting their use to specific conditions, research indicates that sociocultural, behavioural, and economic factors influence antibiotic use that violates recommended guidelines [1,3]. Four major factors relating to the misuse of antibiotics commonly identified globally in the literature include: lack of health literacy regarding AR; ease of access to antibiotics without a prescription; the role of health practitioners in providing prescriptions; and incomplete treatment courses leading to the accumulation of leftover antibiotics [25].

Consumption of leftover antibiotics from earlier prescriptions is one of many antibiotic misuse behaviours that are more likely to occur in older adults who may have limited resources and inadequate health literacy regarding appropriate antibiotic use [20]. Reasons for antibiotic misuse amongst older adults included having more medication than needed, feeling better, experiencing side effects, forgetting to take them, or feeling no difference in symptoms, with over 65% of older adults keeping their leftover antibiotics for themselves [20]. Additional research in the US, UK, Asia, and Africa suggests that over one-third of antibiotic treatment courses/regimens are not adhered to in the general population—50% prematurely cease adherence to antibiotic treatment when improved, and one-third store leftover antibiotics for themselves or others’ future use [26,27].

The use of non-prescription antibiotics has also been influenced by an increased use of technology, with evidence showing that telehealth sessions with a GP are associated with a diminished capability to accurately diagnose and provide appropriate advice about the use of antibiotics [28,29]. The availability of antibiotics being obtained through unauthorized websites, or social media platforms, is also related to technological advances [30]. An Australian investigation of consumer demand for non-prescription medications by Hope et al. (2020) found that 71% of pharmacists were asked by customers for non-prescription access to antibiotics daily or weekly. Up to 75% of pharmacists considered down-scheduling antibiotics to non-prescription status, indicating that increased training in AR-related stewardship policies for pharmacists is required [31].

### 1.4. Theory of Planned Behaviour

**Figure 1:**
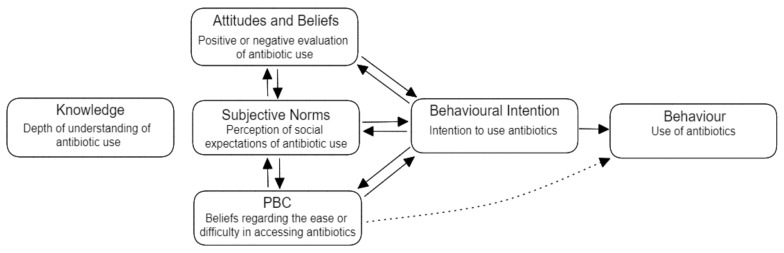
Theory of Planned Behaviour Model, adapted from Ajzen (1991) [35].

Multiple studies have used the Theory of Planned Behaviour (TPB) to try and explain antibiotic use behaviours, with evidence suggesting that it can explain large proportions of previously unexplained variance in these behaviours [4,32]. The TPB suggests three components predict intention to act: perceived behavioural control (PBC), attitudes and beliefs, and subjective norms [33]. Thus, the TPB can be used to identify behavioural, motivational, and social factors that influence intention to misuse antibiotics (Figure 1) [4,34,35,36]. Indeed, Byrne et al. (2019) found that behavioural intention for antibiotic use could be predicted by the three TPB factors, and that knowledge of antibiotic use and AR significantly influenced attitudes and beliefs. The authors developed the Antibiotics Use Questionnaire (AUQ) in consultation with a multidisciplinary panel of experts from fields including psychology, business, and heath. Following the analysis of 293 responses, eighteen items of the questionnaire were retained that reflected the three variables of the TPB, the outcome variable of behaviour, and the covariate of knowledge. The results indicated that antibiotic use behaviour could be significantly explained by each of the variables, and that the TPB model explained 70% of the variance in antibiotic use and misuse.

### 1.5. Aim

The aim of the present study is to replicate the factor structure from Byrne et al. (2019) within an older adult population. Should the factor structure be confirmed, the study then seeks to investigate if the AUQ has the capacity to predict behavioural intentions of antibiotic use and misuse in older adults using TPB constructs. It is hypothesized that knowledge and intention to use antibiotics will be positively associated with the TPB factors, replicating the findings of previous research [4].

## 2. Materials and Methods

### 2.1. Participants and Procedure

To be eligible for the study, participants were required to live independently within their community, be over 70 years of age, and have no known history of cognitive deficits. The criteria of 70 years of age was selected over the usual older adult age-range of 65, as recent research suggests that due to medical and technological advancements in health, older adults are increasingly more independent, have less subjective cognitive decline, and are overall healthier at older ages [37,38,39]. A power analysis for confirmatory factor analysis (CFA) was conducted using the statistical programming language R [40]. The semPower package [41] was used for the calculation, with power set at 0.8, alpha at 0.05, an estimated degree of freedom of 148, and a root mean square error of approximation (RMSEA) of 0.5. This estimated that at least 132 participants were required for our CFA [41].

Recruitment was undertaken via purposive sampling to identify individuals meeting eligibility criteria. A total of 110 participants were recruited within the Darwin community (Northern Australia) and surveyed in-person by the first author (labelled the ‘In-Person’ group). Recruitment took place at local community venues, social events, and local independent living facilities for older adults. Ten participants in this group were given the survey in hard-copy and completed it without the researcher present, returning it via pre-paid mail. Participation was incentivized by entering all participant into a randomly drawn raffle for a $50 Woolworths gift voucher. Eight ‘in-person’ participants were excluded due to incompletion of the survey, leaving 102 persons in this sample. While most ‘in-person’ participants self-completed the survey, 26% of the ‘in-person’ group requested help (labelled as ‘had-help’) to complete it due to issues with reading ability and/or physical impairments such as arthritis. For this group, questions were read aloud to the participant and/or the survey was completed on behalf of the participant by the researcher as they provided their answers.

To increase the sample size in line with our power calculation, an additional 93 participants were recruited using the online crowdsourcing platform M-Turk (labelled ‘M-Turk’ group). This group completed the survey online via the survey platform Qualtrics, with an incentive of $2.00 in Amazon credit for completion of the survey. Bots were controlled for by a forced-response question requiring visual logic ability (‘what is the third word in the following sentence?’). Both groups provided informed consent before participation. This study was conducted in accordance with the National Statement on Ethical Conduct in Human Research and approved by the Charles Darwin University Human Research Ethics Committee (approval no. H22041).

### 2.2. Measures

The Antibiotics Use Questionnaire (AUQ) [4] includes a total of 30 items measured using either dichotomous response options (true or false) or a 4-point Likert scale (strongly agree, agree, disagree, and strongly disagree). Six demographic items are included that measure age, gender (male, female, or other), education (primary school, did not complete secondary school, completed secondary school, TAFE, bachelor’s degree, or Post-Graduate Degree), health training, having friends or family in health work, and postcode. Two items are included for subjective norms (i.e., ‘my friends and family only use antibiotics when prescribed’); four items each are included for behavioural intention, knowledge, PBC, and attitudes and beliefs (i.e., ‘it is my right to ask for antibiotics from my doctor’); and six items randomly selected from the Marlowe–Crowne Social Desirability Scale (SDS) are used to measure the honesty and reliability of answers [42]. The knowledge factor assesses the general understanding of antibiotic use and proximity or accessibility to sources of health information (e.g., ‘antibiotics are needed for the common cold’). The AUQ does not directly measure antibiotic use or behaviour but does measure behavioural intentions related to antibiotic use and misuse (i.e., ‘I would take antibiotics without consulting a doctor’). Please see the Supplementary Materials for a copy of the AUQ (Supplementary Material Measure S1).

### 2.3. Statistical Analysis

All analyses were performed using the statistical software jamovi (version 2.3.9.0) [42]. Initial descriptive analyses and an independent sample *t*-tests were used to compare the In-Person and M-Turk samples. Replicating the strategy used by Byrne et al. (2019), a CFA using orthogonal principal component analysis with varimax rotation was used to confirm the five-factor structure of the AUQ in our sample. Model fit was assessed with several fit metrics including the Comparative Fit Index (CFI), Tucker–Lewis Index (TLI), and RMSEA. CFI and TLI values above 0.95 and RMSEA values below 0.08 were used to indicate good fit to the data [43]. To identify predictors of antibiotic use, an ordinary least-squares regression analysis was used with the behaviour factor (i.e., intention to use antibiotics) of the AUQ as the outcome variable. The predictors included in the model were the three TPB factors (subjective norms, PBC, attitudes and beliefs), demographic variables, and social desirability scores. The knowledge factor was included as a covariate, to replicate prior research [5,34,37].

## 3. Results

### 3.1. Initial Findings

Descriptive analyses found that participants’ mean age was 74.3 years (*SD* = 3.98) and their mean education level was a TAFE qualification (*SD* = 1.40). Social desirability scores showed that 19% (*N* = 39) of all respondents scored ≥5 points (*M* = 3.75, *SD* = 1.03), and these were subsequently excluded from the data, leaving 164 participants in total.

Upon closer analysis, the M-Turk and In-Person groups showed significant mean differences in the education level, age, health training, and health worker in the family factors (Table 1).

Using an Independent Samples T-Test, 14 TPB items out of the 18 also showed significant differences in overall mean scores, with a Mann–Whitney-U test significant in 12 out of 18 items and Shapiro Wilk significant for all items, suggesting a violation of normality (see Table 2).

Due to the significant differences between groups, the results of the CFA were conducted only on the In-Person group, the descriptive statistics of which are reported in Table 3.

The In-Person group included two subgroups: Had Help (*N* = 21), or Self-Completed (*N* = 58). Within the In-Person group, 10 TPB items showed differences in mean scores between subgroups for social desirability, with the subgroup that had help demonstrating a higher mean (*M* = 3.14, *SD* = 0.806) than the group that self-completed the survey (*M* = 3.43, *SD* = 0.507).

### 3.2. CFA of the AUQ

**Figure 2:**
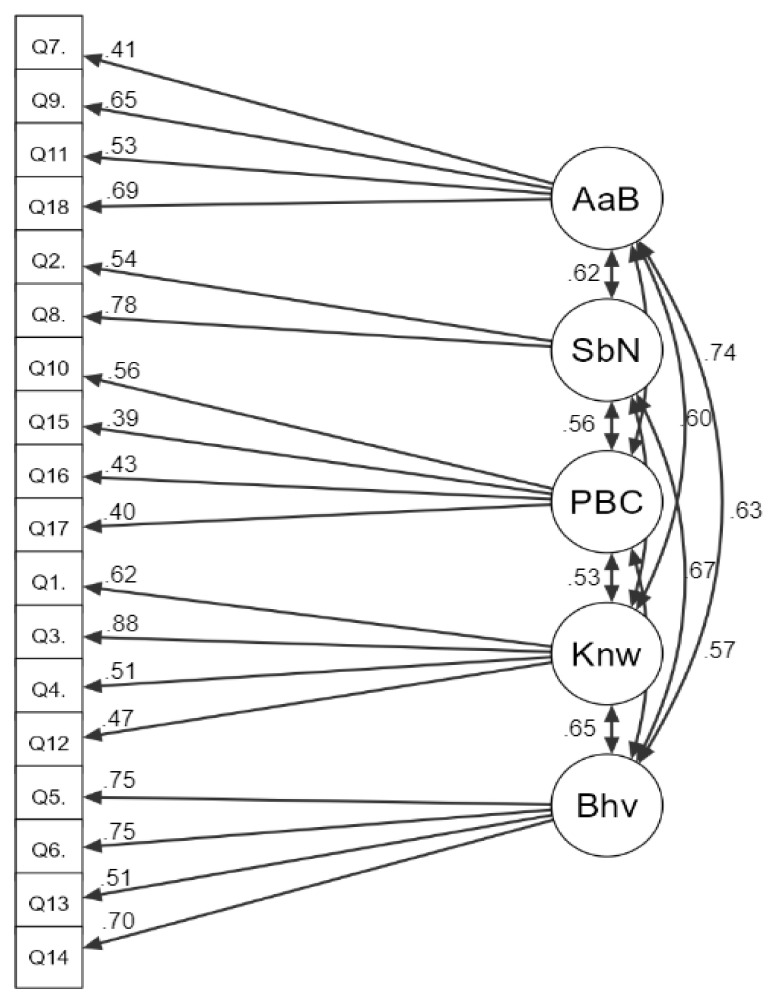
Results of CFA on AUQ factors with standardized parameter estimates. Key: AaB = Attitudes and Beliefs, SbN = Subjective Norms, Knw = Knowledge, Bhv = Behaviour.

Items loading significantly onto relative factors are displayed in Figure 2, showing item loading scores ranging between 0.39 and 0.88 (*p* ≤ 0.05). The fit statistics indicated that the TPB model was a mediocre fit to the data (χ^2^ = 231, *p* ≤ 0.001; CFI = 0.74; TLI = 0.68; RMSEA = 0.10). While the factor structure was confirmed, not all items fit well onto the factor structure, with multiple response items loading significantly onto several factors. Table 4 highlights in red any standardized estimates above 3 to identify items that fit into multiple factors.

Despite exclusion of the three items that loaded significantly on three or more factors, the fit of the factor loadings remained mediocre. Cronbach’s Alpha demonstrated modest but acceptable internal reliability for both groups (M-Turk and In-Person) for all factors (Table 5).

### 3.3. Regression Analyses for the SDS, AUQ Factors and Behaviour

A regression analysis was completed using item means of the outcome variable Behaviour and the AUQ factors within the In-Person group, which found that none of the TPB factors significantly predicted behavioural intention (adjusted R^2^ ≤ 0.3, *p* ≥ 0.05). This was the same for items measuring social desirability (adjusted R^2^ = 0.20, *p* ≥ 0.001). Individually, the item related to healthcare training (β = 0.42, *p* ≤ 0.001), the two PBC items (Q.10 and Q.17), two knowledge items (Q.3 and Q.12), and one attitude and belief item (Q.1) were found to interact with the outcome variable related to behavioural intentions (β = 0.25–0.40, *p* ≤ 0.05). The Shapiro–Wilks test was significant (*p* ≤ 0.71), and VIF indicated low collinearity (VIF ≤ 1.7).

## 4. Discussion

### 4.1. Predictors of Antibiotic Misuse Behaviours

The present study aimed to replicate the factor structure of the Antibiotics Use Questionnaire designed by Byrne et al. [4] in a local, community-based Northern Territorian older adult population. The study additionally aimed to investigate if the AUQ has the capacity to predict behavioural intentions of antibiotic use and misuse in older adults using TPB constructs, with the inclusion of knowledge of AMR as a covariate.

The required sample size proved difficult to obtain, leading to the recruitment of additional OA participants via M-Turk to meet the sample size requirements for a CFA. This resulted in data from a second OA group being analyzed, whose mean demographic and social desirability scores differed significantly from those of the local community-based older adults. As the present study’s aim was to obtain a cohort of verifiably independently living and local older adults whose age and cognitive function was verified through face-to-face administration of the AUQ in public community venues, the M-Turk group was selected for exclusion (in comparison) due to greater relevance of the In-Person group’s demographics to the study’s inclusion criteria.

### 4.2. Comparison with Previous Research

The TPB factor structure from the original study by Byrne et al. (2019) was confirmed in the current study’s local OA population; however, the poor fit of some items to the factor structure indicates that the structure and items of the AUQ may require adaptation to ensure its generalizability to an OA population. This is supported by comparing the 43% of participants in the Byrne et al. [4] study being less than 24 years of age and 58% having reported as having a bachelor’s degree or higher, indicating a significant difference in demographics between the original and current studies’ target populations. These cohort variances may explain differences in item loadings. An alternative explanation of the variance in results may be that the current study’s sample size was limited to 60% of the required number of participants for the CFA. When compared between studies, items that loaded poorly within the factor structure in the current study also showed small factor loadings in the previous study (with standardized estimate coefficients between 0.41 and 0.52), suggesting further research is needed to establish more consistent results using the AUQ [4].

Findings from the regression analysis, hypothesized to replicate Byrne et al.’s (2019) research, found no association between the factors of the TPB, knowledge, or social desirability items and behavioural intentions of antibiotic use, suggesting that in an OA population, the AUQ is unable to reliably predict behavioural intention using factors of the TPB. This contradicts the previous study’s findings, which found that TPB constructs explained 70% of the variance in behavioural intentions related to antibiotic use [5]. Comparatively, Byrne et al. [4] found that demographic variables did not significantly predict behavioural intention in their sample, whereas healthcare training showed a significant interaction with behavioural intention in the current study—although similar in both studies, no other demographic variable was significantly correlated with behavioural intention. The lack of correlation between the TPB factors of the AUQ and antibiotic use behaviour in the current study may again be explained by the small sample size, or the differences in population demographics between the two studies that potentially render the current version of the AUQ unsuitable for use with older adults [4]. Contradictory to the current study and the study conducted by Byrne et al. [4], other research has found significant predictors of antibiotic use in demographic variables. For example, research has found that having a healthcare worker as a friend or family member was associated with increased antibiotic misuse [44,45,46]. These discrepancies in the findings further suggest the potential generalizability and/or effect size issues with the population sample of the current and the previous replicated study [4].

### 4.3. Older Adults and TPB Factors

Factors of the TPB model must be recognised as defining different areas of behavioural intention for older adults, whose social, emotional, and economical contexts can differ substantially from the younger populations [38]. In particular, PBC for older adults constitutes a different factor within the TPB than for the younger population: physiological impairment is a significant barrier to PBC and health-related self-efficacy, as conditions such as dementia, arthritis, heart disease, hearing loss, and diabetes largely affect mental health, mobility, diet and nutrition, memory, and sleep [47,48,49]. These issues can impact the independence of older adults, preventing them from driving, self-care, and essential self-maintenance behaviours such as regular medication adherence [50]. Psychological issues such as depression, anxiety, and prolonged grief are also common in older adults due to these limiting and significant physiological, social, and environmental changes in themselves and their relationships with loved ones [51]. Additionally, ageism reported in research experienced by older adults in GP clinics and other healthcare settings, such as pharmacies and hospitals, is suggested to affect the self-efficacy of older adults in being able to communicate their needs effectively, and to feel supported and informed by healthcare professionals [52]. PBC in accessing and using antibiotics with or without a prescription, and adherence to appropriate guidelines for antibiotics, are likely to be impacted by each of these factors for older adults, and in turn, affect attitudes and beliefs about their antibiotic use, as well as subjective norms when relating to others.

The concept that PBC has greater influence on behavioural intention for older adults contradicts previous research utilizing the TPB, which typically weights the TPB constructs equally. However, it has been suggested that there is potential that PBC may serve as a moderating variable for attitudes and beliefs and subjective norms [34,37,53]. A study by La Barbera and colleagues [36] found that levels of PBC were positively associated with attitudes and beliefs, and both negatively and positively associated with subjective norms. This suggestion may explain the results in the original study by Byrne et al. [4], which found that subjective norms had the weakest internal consistency compared to other factors and contained only two items. This is supported by findings from Castanier et al. (2013), who found that higher PBC was correlated with lower subjective norms (i.e., people who felt more in control were less likely to be influenced by peer pressure) [54]. These findings may relate to the current sample of older adults, in that the participants selected were assumed to have higher-than-average PBC for their age group due to their active, engaged, and independent participation in social clubs and events. Additionally, Sussman and Gifford (2019) suggest the TPB can be interpreted as having a reverse-causal relationship, with behaviour being influenced by the three base factors [53]. These potentially multidirectional interactions between behavioural intention, knowledge, and PBC for older adults and health beliefs may provide an additional alternative explanation for the differences in factor structure loadings between the current study and the previous study by Byrne et al. (2019) [4]. More broadly speaking, it may also provide further evidence of the complexities of older adults’ choices and experiences regarding antibiotic use behaviours that must be considered when constructing or adapting health behaviour intervention measures and strategies for this population.

### 4.4. Strengths and Limitations

The current study benefited from the inclusion of a widely representative sample of OA participants from all areas within the local community and from a wide range of cultural backgrounds represented in Australia’s Northern Territory. Limitations of this study include the challenges with obtaining an adequate sample size due to the low numbers of independent and accessible local Northern-Territorian older adults. Furthermore, the differences between the data collected through M-Turk and in person resulted in the exclusion of participants recruited online, resulting in a smaller sample size being used than what was required for a CFA.

Similarly, there was a potential for bias due to differences in cognitive ability, as the current study did not include controls for diagnosed cognitive deficits or decline. Differentiating these from naturally occurring, age-related subjective cognitive decline is recognised as a complex issue in self-reported health behaviour research involving older adults [55,56]; however, the inclusion of appropriate measures was beyond the scope of the current study. Issues with validity related to the social desirability also arose, given that the measure was self-reported, leading to a higher likelihood of falsified responses [57] within the In-Person group. The subgroup that was assisted with their survey responses demonstrated higher scores for social desirability bias than the group that completed the survey themselves, indicating that the assistance of the researcher likely resulted in increased social desirability. Therefore, despite the benefit of increased survey accessibility due to researcher assistance in completion of the survey, this assistance may have skewed the data.

Lastly, the differences in perceived behavioural control compared to actual behavioural control limits the ability to predict antibiotic use behaviour from intentions. It should also be noted that intention to act on a behaviour does not guarantee the behaviour will be acted upon (for example, people are unlikely to use antibiotics unless they are sick enough or feel they need them, regardless of their intentions).

## 5. Conclusions

The primary aim of the study was to replicate the factor structure from Byrne et al. (2019) [4] within an older adult population. Results from the current study show that this factor structure is indeed confirmed. Despite these results, limitations due to sample size and accessibility restricted the generalizability and validity of results, and no correlation was found between behavioural intention and antibiotic use behaviours. Further research is required to adapt AUQ items specifically for older adults and confirm this factor structure in OA populations with a larger sample size, in-person recruitment, and more accessible and efficient AUQ delivery. More accessible methods of conducting the survey are recommended for this age group, such as assisted electronic delivery via tablet, where questions are pre-recorded to be played out loud if needed, which would control for social desirability bias found in the current study. Additionally, accounting for the differences in PBC in the OA population is suggested when adapting items to better measure antibiotic use behaviours in older adults. Finally, future research involving older adults would benefit from measures controlling for cognitive decline, such as a test of grip strength, which has been shown to have good predictive validity, and the addition to the AUQ of an item requesting an indication of severity of self-identified subjective cognitive decline [58]. Overall, these results suggest that the AUQ has the potential to become a valuable tool to measure behavioural intentions for antibiotic use in older adults and supports research that suggests that age-specific training and transparency regarding information on AMR is required by health service providers when treating older adults.

## Figures and Tables

**Table 1:** Mean Differences Between Groups.

	Survey Platform	N	Mean	SD	T-Statistic	*p*-Value
Education	In-Person	79	3.49	1.395	−9.69	<0.001
	M-Turk	85	5.19	0.779		
Healthcare Training	In-Person	79	1.80	0.404	10.09	<0.001
	M-Turk	85	1.18	0.383		
Healthcare Worker in Family	In-Person	79	1.47	0.502	5.61	<0.001
	M-Turk	85	1.11	0.310		
Age	In-Person	79	76.65	4.139	8.90	<0.001
	M-Turk	85	72.09	2.175		

**Table 2:** Independent Samples T-Test—Mann–Whitney U For TPB Items.

		Statistic	*p*
Q1. Abs Reduce Cold Symptoms	Mann-Whitney U	3275	0.760
Q2. Friends & Family Follow AB Recommendations	Mann-Whitney U	824	<0.001
Q3. Abs Are Needed for Colds	Mann-Whitney U	3350	0.978
Q4. Abs Can Have Negative Side Effects	Mann-Whitney U	855	<0.001
Q5. I Use Abs Without Dr. Consultation	Mann-Whitney U	3162	0.490
Q6. I Use Leftover Abs	Mann-Whitney U	3217	0.619
Q7. It’s My Right to Ask for ABs	Mann-Whitney U	819	<0.001
Q8. Friends & Family	Mann-Whitney U	1037	<0.001
Q.9 Know When I Need AB’s	Mann-Whitney U	2158	<0.001
Q10. Use of ABs Without Prescription is Common	Mann-Whitney U	1952	<0.001
Q11. Confident to Ask for AB’s	Mann-Whitney U	995	<0.001
Q12. Abs Will be Less Effective in Future	Mann-Whitney U	970	<0.001
Q13. I Consult Dr. Prior to Taking ABs	Mann-Whitney U	417	<0.001
Q14. I Keep Leftover ABs	Mann-Whitney U	2878	0.090
Q15. Easily Get Abs from Dr.	Mann-Whitney U	1465	<0.001
Q16. Easily Get Abs Online	Mann-Whitney U	2717	0.018
Q17. Easily Get Abs Family	Mann-Whitney U	3147	0.438
Q18. Expect Abs from Dr.	Mann-Whitney U	2553	0.004

**Table 3:** In-Person Group Descriptive Statistics for Demographics and TPB.

	Self-Completed or Had Help	Mean	SD
Gender	Self-Completed	1.67	0.482
	Had Help	1.57	0.535
Education	Self-Completed	3.71	1.654
	Had Help	2.57	0.976
Age	Self-Completed	78.17	3.435
	Had Help	80.43	5.593
Health Training	Self-Completed	1.83	0.387
	Had Help	1.86	0.378
Health Worker in Family	Self-Completed	1.50	0.511
	Had Help	1.71	0.488
Behaviour	Self-Completed	2.14	0.410
	Had Help	2.54	0.419
Social Desirability Scale	Self-Completed	5.13	0.338
	Had Help	5.00	0.000
Knowledge	Self-Completed	2.58	0.319
	Had Help	2.64	0.378
Attitudes and Beliefs	Self-Completed	2.54	0.588
	Had Help	2.79	0.585
Subjective Norms	Self-Completed	2.98	0.454
	Had Help	2.86	0.244
Perceived Behavioural Control	Self-Completed	1.82	0.486
	Had Help	2.04	0.304

**Table 4:** CFA Modification Indices for In-Person Group Factor Loadings.

	Attitudes & Beliefs	Subjective Norms	Perceived Behavioural Control	Knowledge	Behaviour
Q7. It’s My Right to Ask for ABs		0.508	0.227	2.207	0.048
Q9. Know When I Need ABs		1.209	3.894	0.301	1.371
Q11. Confident to Ask for ABs		5.325	7.097	0.817	4.715
Q18. Expect Abs form Dr.		5.955	20.565	5.336	10.053
Q2. Friends & Family Follow AB Recommendations	4.164		1.178	1.368	3.140
Q8. Friends & Family Only Use Prescribed ABs	4.164		1.178	1.368	3.140
Q10. Use of Abs Without Prescription is Common	0.798	3.309		1.885	3.664
Q15. Easily Get Abs from Dr.	6.717	0.014		0.001	9.237
Q16. Easily Get Abs Online	10.109	5.341		5.631	3.962
Q17. Easily Get Abs Family	0.087	3.224		0.581	7.848
Q1. Abs Reduce Cold Symptoms	0.002	2.682	0.664		2.476
Q3. Abs Are Needed for Colds	0.907	0.174	0.180		0.375
Q4. Abs Can Have Negative Side Effects	3.221	0.007	0.002		0.025
Q12. Abs Will be Less Effective in Future	0.235	2.674	3.329		7.758
Q5. I Use Abs Without Dr. Consultation	0.018	0.006	1.675	5.455	
Q6. I Use Leftover ABs	1.386	0.452	0.064	1.434	
Q13. I Consult Dr. Prior to Taking ABs	0.059	0.233	0.609	0.048	
Q14. I Keep Leftover ABs	1.693	0.109	1.179	1.893	

**Table 5:** Reliability and Descriptive Statistics for TPB Scales.

	Survey Platform	Mean	SD	Cronbach’s α
Behaviour	In-Person	2.76	0.56	0.68
	M-Turk	2.32	0.69	0.77
Knowledge	In-Person	3.03	0.53	0.70
	M-Turk	2.73	0.54	0.53
Perceived Behavioural Control	In-Person	2.02	0.40	0.48
	M-Turk	2.03	0.53	0.59
Subjective Norms	In-Person	2.83	0.55	0.59
	M-Turk	2.86	0.76	0.75
Attitudes & Beliefs	In-Person	2.76	0.56	0.68
	M-Turk	2.32	0.69	0.77

## Data Availability

The data presented in this study are available on request from the corresponding author. The data are not publicly available due to the potential for participant identification.

## Supplementary Materials

The following supporting information can be downloaded at: https://www.mdpi.com/article/10.3390/antibiotics12040718/s1. Measure S1: The Antibiotic Use Questionnaire.
