# Supplementary material for: Antibiotic Misuse Behaviours of Older People: Confirmation of the Factor Structure of the Antibiotic Use Questionnaire
Source: Antibiotics (Basel). 2023 Apr 6;12(4):718. doi: 10.3390/antibiotics12040718 (PMC10135189; doi:10.3390/antibiotics12040718)
Supplement: Supplementary file 1 [file antibiotics-12-00718-s001.zip › antibiotics-2140508-supplementary.pdf]

Measure S1 – Antibiotic Use Questionnaire

1. What is your age?

____

2. What is your gender?

- ☐ Male
- ☐ Female
- ☐ Other

3. What is the highest level of education you have completed?

- ☐ Primary School
- ☐ Secondary School, did not complete year 12
- ☐ Secondary School, completed year 12
- ☐ TAFE
- ☐ Bachelor's Degree
- ☐ Postgraduate Degree, i.e. Masters, PhD

4. What is your post code?

____

5. Are you trained in health-related fields?

- ☐ Yes
- ☐ No

6. Is there a healthcare worker in your family or friend group?

- ☐ Yes
- ☐ No

7. Antibiotics will reduce my cold symptoms

- ☐ Strongly Disagree
- ☐ Disagree
- ☐ Agree
- ☐ Strongly Agree

8. My friends and family follow recommendations for antibiotic use

- ☐ Strongly Disagree
- ☐ Disagree
- ☐ Agree
- ☐ Strongly Agree

9. Antibiotics are needed for the common cold

- ☐ Strongly Disagree
- ☐ Disagree
- ☐ Agree
- ☐ Strongly Agree

10. I am always courteous, even to people who are disagreeable

- ☐ True
- ☐ False

11. Antibiotics may have negative side effects

- ☐ Strongly Disagree

- Disagree
- Agree
- Strongly Agree

12. There have been times when I felt like rebelling against people in authority even though I knew they were right

- True
- False

13. I would take antibiotics without consulting a doctor

- Strongly Disagree
- Disagree
- Agree
- Strongly Agree

14. I use leftover or unused antibiotics or scripts

- Strongly Disagree
- Disagree
- Agree
- Strongly Agree

15. No matter who I'm talking to, I'm always a good listener

- True
- False

16. It is my right to ask for an antibiotic from my doctor

- Strongly Disagree
- Disagree
- Agree
- Strongly Agree

17. My friends and family only use antibiotics when prescribed

- Strongly Disagree
- Disagree
- Agree
- Strongly Agree

18. I'm always willing to admit it when I make a mistake

- True
- False

19. I know I need antibiotics before I see my doctor

- Strongly Disagree
- Disagree
- Agree
- Strongly Agree

20. In my community, it is common to use antibiotics without a prescription

- Strongly Disagree
- Disagree
- Agree
- Strongly Agree

21. I feel confident to ask for antibiotics when I need them

- Strongly Disagree

- Disagree
- Agree
- Strongly Agree

22. I have never deliberately said something that hurt someone's feelings

- True
- False

23. Antibiotics are less likely to work in the future

- Strongly Disagree
- Disagree
- Agree
- Strongly Agree

24. I consult with my doctor prior to taking antibiotics

- Strongly Disagree
- Disagree
- Agree
- Strongly Agree

25. There have been times when I was quite jealous of the good fortune of others

- True
- False

26. I keep leftover or unused antibiotics or scripts

- Strongly Disagree
- Disagree
- Agree
- Strongly Agree

27. I could easily get antibiotics from a doctor

- Extremely Difficult
- Difficult
- Easy
- Extremely Easy

28. I could easily get antibiotics online

- Extremely Difficult
- Difficult
- Easy
- Extremely Easy

29. I could easily get antibiotics from my family, a friend/household

- Extremely Difficult
- Difficult
- Easy
- Extremely Easy

30. By the time I am sick enough to see my doctor, I expect a prescription of antibiotics

- Strongly Disagree
- Disagree
- Agree
- Strongly Agree
